# Enostosis-related epilepsy

**DOI:** 10.1259/bjrcr.20200152

**Published:** 2021-01-07

**Authors:** Jorge Vaz Lourenço, Joana Coelho, Andrea Salgueiro

**Affiliations:** 1 Department of Internal Medicine, Centro Hospitalar Universitário de Coimbra, Coimbra, Portugal; 2 Department of Emergency, Centro Hospitalar Universitário de Coimbra, Coimbra, Portugal

## Abstract

Unexpected bone lesions of the skull present a common dilemma, where radiological appearance and patient’s clinical background are crucial to avoid misdiagnosis.

Enostosis are benign sclerotic bone lesions; its aetiology is still unknown and its management is usually conservative, with good prognosis. Most of these lesions are asymptomatic and neurological involvement is rare. We present the first report of enostosis-related epilepsy.

## Clinical presentation

A 64-year-old male was referred to our emergency department (ED) presenting with altered state of consciousness. He was found unconscious by his daughter at home who also described a “generalized shaking”. She denied signs of trauma, sphincter incontinence and confirmed that he was fine the day before. By the time the patient got to the hospital, he spontaneously recovered and had no memories of the event. There was no history of previous events, drug or alcohol abuse. His past medical history included arterial hypertension with long-term use of perindopril 5 mg bid.

**Figure 1.**
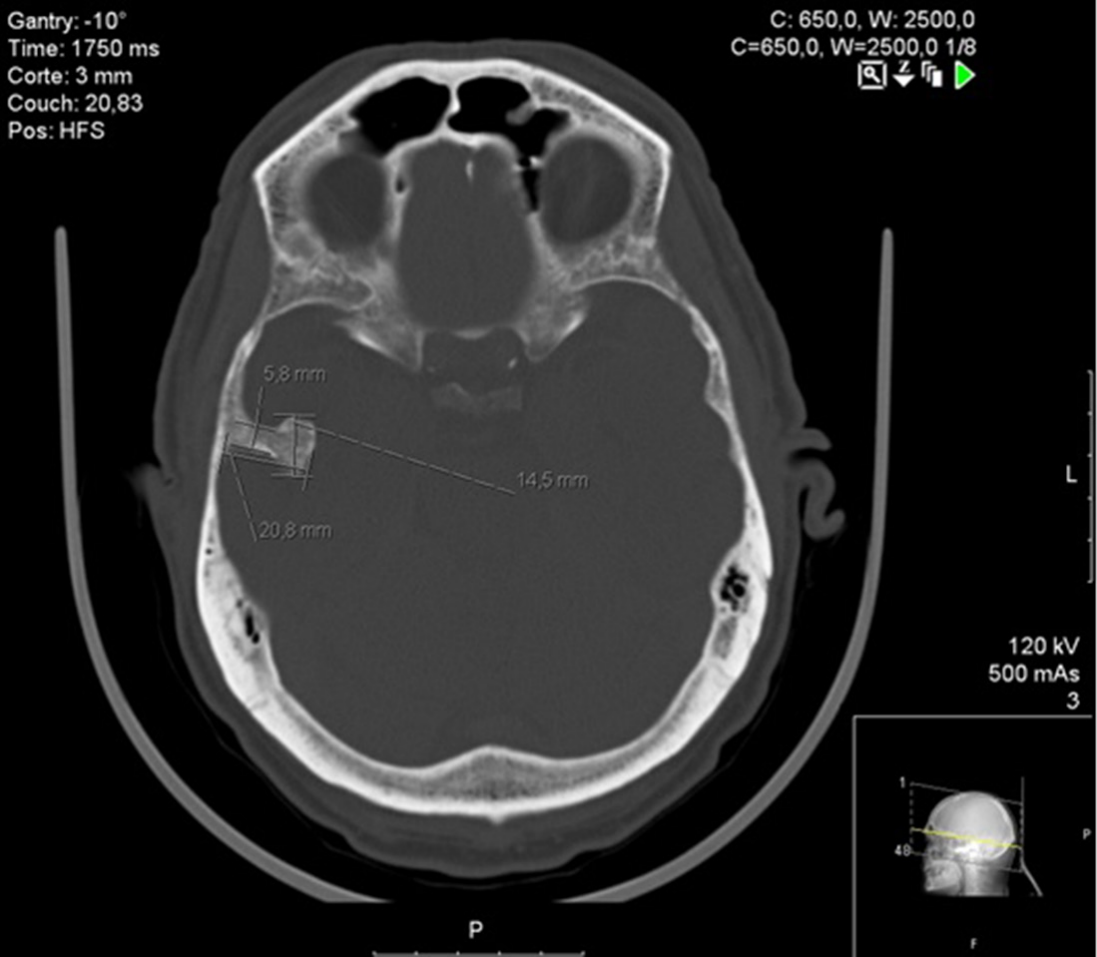
Right temporal enostosic lesion on non-contrast brain CT (bone image).

**Figure 2.**
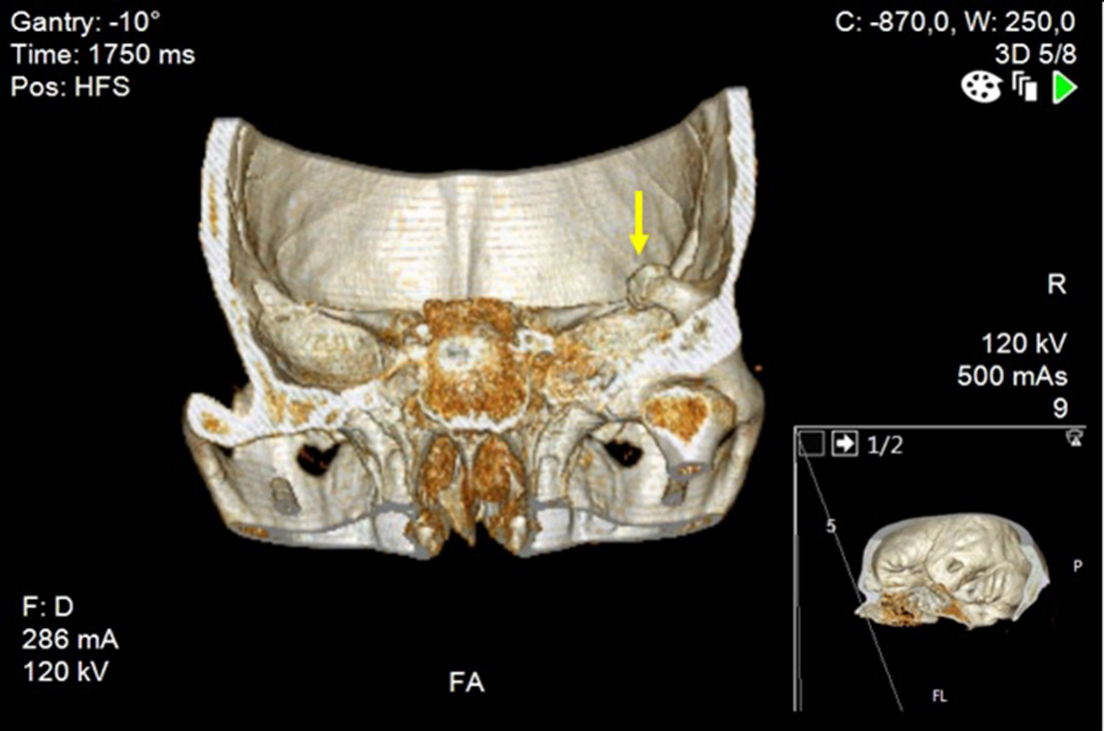
3D reconstruction-CT of the enostosic lesion (yellow arrow)

**Figure 3.**
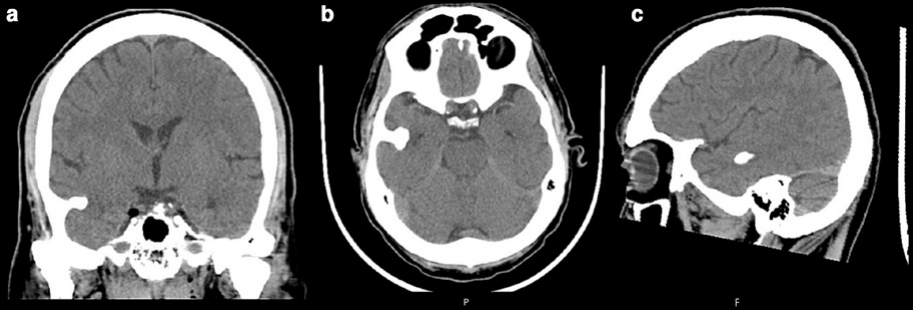
Brain CT showing no parenchymal compromise in coronal (**A**), axial (**B**) sagittal and (**C**) planes

In the ED, he was hemodynamically stable, afebrile and had a normal neurologic exam. His arterial blood gas test confirmed normoglycemia and excluded significant acid-base or ionic disturbances. The rest of his blood analysis showed only discrete serum CK elevation (762 U l^−1^ for a reference range of <145 U l^−1^), with normal renal and hepatic function tests as well as preserved complete blood count. During his time in ED, he had a brain CT that detected a tubular 20.8 × 14.5 mm enostosic formation in the right temporal fossa with resulting mass effect on the adjacent temporal lobe (Figures 1 and 2). There was no ventricular involvement, edema, structural deviations, concussion or hemorrhage. (Figure 3). Subsequently, he suddenly became stuporous and unresponsive to verbal or tactile stimuli.

**Figure 4.**
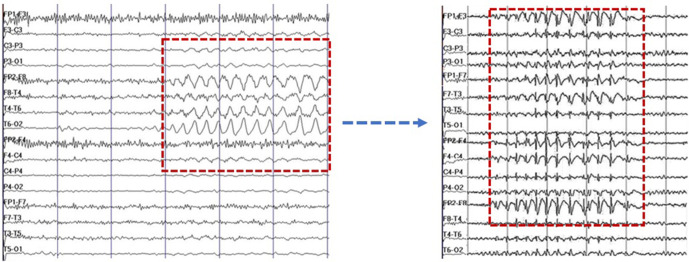
Patient’s EEG shows generalized epileptiform activity derived from right temporal lobe

An electroencephalogram (EEG) was performed that detected generalized epileptiform activity derived from right temporal lobe (Figure 4).

## Treatment

The patient recovered after treatment with levetiracetam and was then observed by neurosurgery who decided to evaluate the case later on. The patient was discharged with long term use of levetiracetam 500 mg bid and is pending final decision on whether surgical treatment should be performed.

## Discussion

Enostosis represents a slow-growing benign and highly mineralized bone lesion derived from cancellous bone.^1^ The pathogenesis is uncertain, but it is thought to be congenital or developmental in nature, where self-limiting osteoclastic dysfunction plays the main role during bone remodelling.^1^ Its real prevalence remains unknown; it seems to appear in childhood, equally affecting both genders.^2^ Proximal long bones and pelvis are most affected; they are rarely seen associated with cranial bones.^1,2^

The radiological appearance on radiograph, CT or MRI could be seen as small round and dense bone within the medullary space, sometimes with classic radiating spicules at the margins that blend with the surrounding trabeculae.^3^

The main differential diagnosis consists of osteoid osteoma, osteosarcoma and osteoblastic metastasis.^4,5^

The management of these lesions is usually conservative and reveals good prognosis.^6^

Enostosis are mainly an incidentaloma; if present, symptoms are associated with their anatomic location.^6^ Surgical removal could be necessary in cases of refractory and function-limiting lesions, due to compression of surrounding structures.^1,6^ There is insufficient data regarding neurological involvement and this is to our knowledge the first reported case of enostosis-related epilepsy.

## Learning points

1. Enostosis is often incidental, they represent slow growing bone lesions and most of related symptoms are due to involvement of surrounding structures.
2. Neurological involvement is rare. Those with disabling lesions require surgical treatment.
